# From free text to structured genomics: future-proofing cancer registries for precision oncology

**DOI:** 10.1016/j.esmorw.2026.100755

**Published:** 2026-09-17

**Authors:** J. Zohner, C. Kuhl, K. Schmid, S.-Z. Kim-Wanner, C. Kujawa, D. Amsel

**Affiliations:** 1 Department of Medicine, Data Integration Center, Giessen University Hospital, Giessen, Germany; 2 Hessian Cancer Registry Department, Trusted Office, Hessian Medical Association, Frankfurt am Main, Germany; 3 Department of Medicine, Institute for Neuropathology, Justus-Liebig-University Giessen, Giessen, Germany; 4 Hessian Cancer Registry Department, Hessian State Office for Health and Care, Frankfurt am Main, Germany

**Keywords:** cancer registries, interoperability, molecular pathology, precision oncology, real-world data, structured genomic data

## Abstract

Molecular pathology has become central to precision oncology, but German cancer registry data structures were designed for simpler molecular findings. This study argues that registries need a minimal, structured, and interoperable representation of molecular variants that remains compatible with data minimization and existing reporting infrastructures. Using the German oncology core dataset (nationally standardized oncological core dataset for cancer reporting—einheitlicher onkologischer Basisdatensatz [oBDS]) as an example, we outline conceptual principles for a backward-compatible molecular data structure and propose a future agenda for consensus-building, pilot implementation, and impact evaluation.

## Highlights

• Current German cancer registries poorly capture molecular oncology complexity.
• We propose a backward-compatible oBDS extension for structured variant data.
• Key HGVS parameters are encoded within existing registry fields, with no schema change.
• Structured reporting boosts machine readability, interoperability, and reuse.
• The concept future-proofs registries and strengthens real-world evidence.

## Introduction—molecular oncology outgrows cancer registry structures

Cancer registries are central infrastructures for population-based surveillance, quality assurance, research, and real-world evidence generation. In Germany, the 2013 Cancer Screening and Registration Act and the nationally standardized oncological core dataset have substantially strengthened the coverage and comparability of clinical cancer registration.^1^^,^^2^ However, these infrastructures now face a new challenge: molecular oncology has evolved faster than many registry data models.

Molecular pathology has evolved into a central pillar of personalized medicine. Early single-gene, hotspot-based testing yielded binary mutated/wild-type results, whereas targeted panels and whole-exome/genome sequencing now generate high-dimensional data with broad variant spectra and contextual dependencies.^3^, ^4^, ^5^, ^6^, ^7^, ^8^, ^9^, ^10^, ^11^ This creates an inherent need for standardized variant annotation to support interpretability, compatibility, and reuse.^5^^,^^12^

These developments create structural tensions within cancer registries: molecular complexity and granularity have increased, while registry data models have remained oriented toward well-defined hotspot variants with known wild-type or mutated status.^2^^,^^13^ As a result, current models may limit the structured, machine-readable, and interoperable representation of molecular information, particularly for longitudinal analyses, cross-institutional comparison, or linkage with emerging real-world data infrastructures.^13^^,^^14^ This creates a registry-policy challenge: molecular data must be structured enough to remain interpretable and reusable, while respecting data minimization, which is especially consequential for genomic data given their sensitivity and reidentifiability.^15^, ^16^, ^17^ The central question is therefore not whether registries should collect ever more comprehensive genomic data but how to define a minimal, structured, interoperable representation compatible with their public health mandate, legal responsibilities, and existing infrastructure. This study addresses this question using the German oncological core dataset as an example of a national registry standard facing the transition from classical tumor documentation toward precision oncology.

## Structural limitations of the current nationally standardized oncological core dataset for cancer reporting (oBDS)

**Figure 1:**
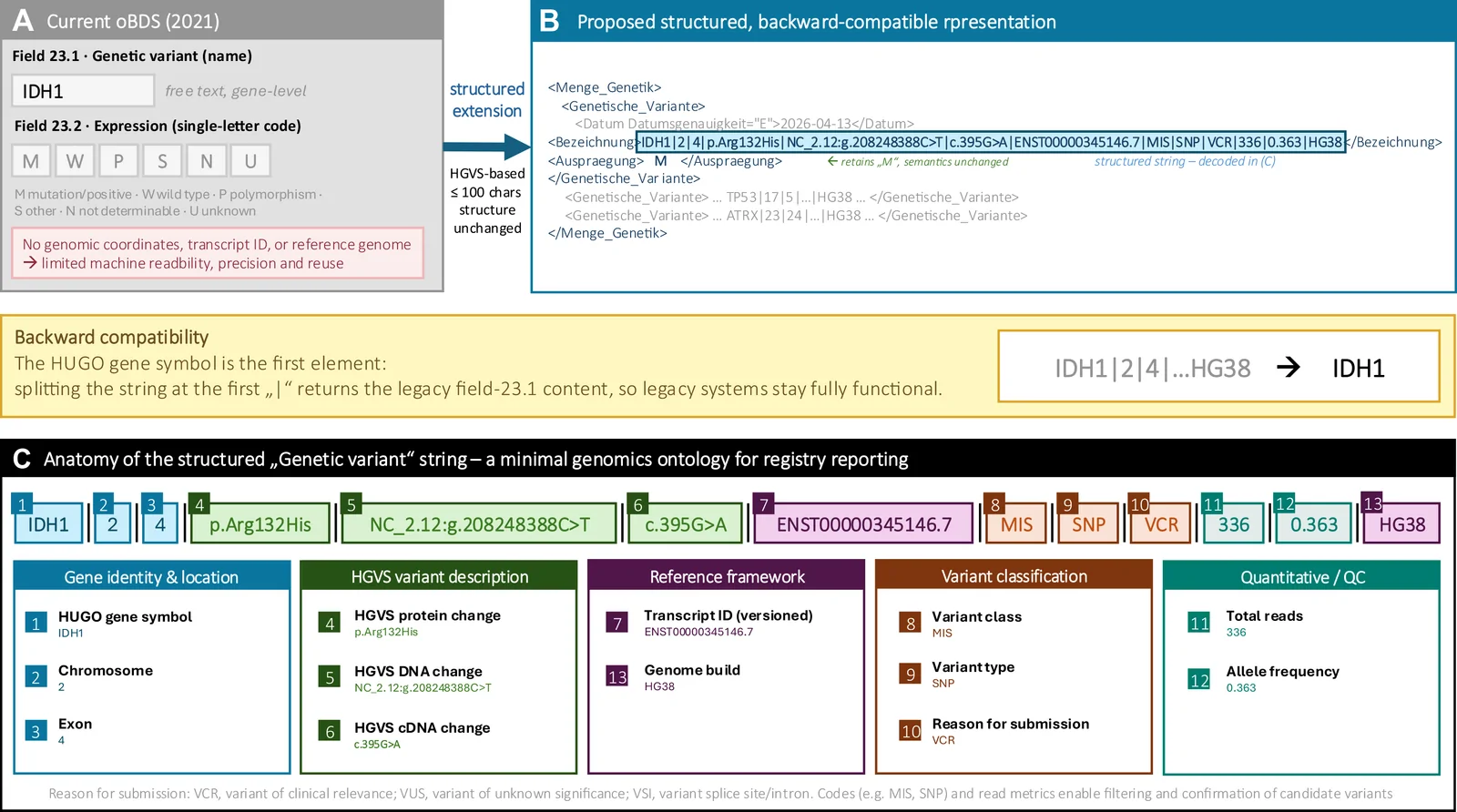
**(A) Current representation (oBDS 2021).** Molecular findings are captured in two fields: field 23.1 (*‘*Genetic variant*’*) holds a free text, gene-level identifier (e.g. isocitrate dehydrogenase 1 [IDH1]), and field 23.2 (‘Expression’) a single-letter status code (M, mutation/positive; W, wild type; P, polymorphism; S, other; N, not determinable; U, unknown). This representation lacks genomic coordinates, transcript identifiers, and reference genome information, limiting machine readability, semantic precision, and reuse. (B) Proposed structured, backward-compatible representation. The existing oBDS XML container (Menge_Genetik → Genetische_Variante) is retained without altering the formal dataset structure and the expression field (Auspraegung) remains ‘M’, preserving existing semantics. All additional parameters are encoded as a single pipe-delimited string within the existing ‘Genetic variant’ field (Bezeichnung), respecting the 100-character constraint. Because the HUGO gene symbol is the first element, splitting the string at the first delimiter returns exactly the legacy field 23.1 content (IDH1) so systems that do not parse the extended syntax remain fully functional. (C) Anatomy of the structured string. The 13 ordered, machine-readable elements are illustrated for the example variant IDH1…HG38 and grouped into five conceptual categories that constitute the proposed genomics ontology: gene identity and location (HUGO gene symbol, chromosome, and exon); HGVS-based variant description at the protein, DNA, and coding DNA levels; the reference framework that renders each call unambiguous (versioned transcript identifier and reference genome build); variant classification (variant class, variant type, and reason for submission—VCR, variant of clinical relevance; VUS, variant of unknown significance; VSI, variant splice site or intron); and quantitative quality metrics (total read depth and variant allele frequency). Together, the panels show how a minimal, ontology-based extension converts unstructured, gene-level entries into precise, interoperable, and reusable variant records while preserving full backward compatibility with the current oBDS. HGVS, Human Genome Variation Society; HUGO, Human Genome Organisation.

The current nationally standardized oncological core dataset for cancer reporting (einheitlicher onkologischer Basisdatensatz [oBDS], version 2021 [GNCR, Lübeck, Germany; ADT, Berlin, Germany; Plattform 65c, Lübeck, Germany; published in the German Federal Gazette]) provides only limited granularity for the structured representation of molecular genetic findings. Although molecular information is formally included, the dataset was not designed to capture the increasing contextual dependencies, complexity, and granularity of molecular oncology. As a result, molecular information is often reduced from structured diagnostic systems into simplified registry fields or free text, limiting downstream use for harmonization, quality assurance, and research (Figure 1).

A central limitation is the lack of machine readability and semantic precision. Without unambiguous identifiers, versions, or genomic coordinates, identical variants may be represented differently across institutions, creating inconsistencies that are difficult to resolve retrospectively. Without explicit transcript identifiers and versions, genomic coordinates, and reference genome builds (e.g. human genome (HG)19/Genome Reference Consortium Human Genome Build (GRCh)37, HG38/GRCh38, or Telomer-to-Telomer [T2T]), molecular findings may not be reliably mapped to underlying DNA alterations or reinterpreted as knowledge evolves, compromising long-term usability for harmonization or integration with external datasets.^3^^,^^4^^,^^17^, ^18^, ^19^ The dataset is also of limited scalability. Oriented toward single-gene or hotspot-based testing, it does not readily accommodate the high-dimensional output of gene panels or whole-exome/genome sequencing nor the integration of emerging biomarkers or updated nomenclature without *ad hoc* solutions. Some legacy categories, such as explicit wild-type notation, also become less informative in panel-based sequencing, in which the absence of a reported variant may reflect a true negative, a reporting threshold, or missing information (Figure 1).

Implicitly, the current oBDS assumes that free-text documentation can represent complex genomic information. As the number of variants reported per case grows, this assumption scales poorly. Free text may support immediate clinical interpretation but is less suited to longitudinal analyses, cross-institutional comparison, automated quality assurance, or secondary use in research.

## A pragmatic extension: conceptual principles for a backward-compatible molecular data model in cancer registries

The oncological core dataset is a nationally established standard embedded in legal, technical, and organizational infrastructures. We therefore argue for evolutionary extension rather than replacement, preserving compatibility with existing registry, legal, and software structures. Several design principles are relevant. Firstly, backward compatibility so that previously collected data remain usable and adaptations do not require fundamental system changes. Secondly, data minimization: molecular data collection should focus on parameters that are necessary, interpretable, and reusable for defined registry purposes, rather than comprehensive genomic annotation. Thirdly, syntactic clarity, to reduce ambiguity across reporting institutions. Finally, interoperability with laboratory information systems and alignment with international nomenclature standards such as the Human Genome Variation Society (HGVS) are central to consistent data exchange and long-term usability.

To illustrate how more structured molecular documentation could be approached within existing registry constraints, we outline a conceptual model that builds on the existing fields 23.1 (‘Genetic variant’) and 23.2 (‘Genetic expression’) without altering the formal structure of the dataset. A key registry constraint is the 100-character limit of the relevant field, which illustrates the need for a compact and standardized representation.

In the illustrative model, the field ‘Genetic expression’ (item 23.2) would remain documented in its established form using the single-letter code ‘M’ for a reported mutation. In next-generation sequencing, the absence of a reported mutation should not be read as an explicit wild-type result, as it may reflect reporting thresholds, panel composition, or missing information; the existing semantics should therefore be preserved in a first step.

In this illustrative model, selected parameters relevant for molecular interpretation could be represented in a structured format within the field ‘Genetic variant’ (item 23.1). For illustrative purposes, a string-based syntax could be envisaged in which individual elements are separated by pipe characters (‘|’) and arranged in a fixed order. Such an encoding system could, in principle, capture selected parameters in a consistent and compact form while respecting existing character limitations. Importantly, placing the gene symbol first could preserve backward readability. Systems that do not process the extended syntax could still identify the gene before the first delimiter (Figure 1).

As a possible future extension, a controlled reason-for-submission category could be discussed to distinguish different reporting contexts. This could be VSI (variant splice site or intron), VCR (variant of clinical relevance), and VUS (variant of unknown significance). Such a category could help distinguish confirmed variants from candidate findings but would require governance, consensus definitions, and validation before routine use.

## Future implementation agenda

Future implementation of structured molecular registry documentation will require a stepwise and multistakeholder process. Firstly, the proposed conceptual model should be discussed with cancer registries, molecular pathology laboratories, clinical stakeholders, software vendors, data protection experts, and regulatory bodies. Such a consensus process is needed to define which molecular parameters are necessary and proportionate for registry purposes and which should remain within laboratory or clinical systems.

Secondly, the proposed parameter set should undergo legal and governance review, particularly with regard to data minimization, purpose limitation, and the sensitivity of genomic data. This step is essential to ensure that registry-compatible molecular documentation improves interpretability without expanding data collection beyond justified registry purposes.

Thirdly, technical feasibility should be evaluated in pilot implementations. This should include compatibility testing with existing registry software, laboratory information systems, and data exchange formats. Particular attention should be paid to character limits, parser readability, error handling, backward readability, and the preservation of existing oBDS semantics.

Fourthly, the representability of real-world molecular findings should be assessed using molecular pathology reports from different tumor entities, sequencing panels, and reporting institutions. Such studies should evaluate how often relevant variants can be represented within the proposed structure, which information is lost or ambiguous, and which variant types require additional modeling.

Finally, future studies should assess clinical, research, and operational impact. Relevant outcomes may include data completeness, reduction of ambiguous variant documentation, improved registry query ability, feasibility of cross-institutional harmonization, and usability for quality assurance and real-world evidence generation. Only after such evaluation should routine implementation or formal integration into national registry standards be considered.

## Conclusion: Toward future-ready cancer registries

This study argues that a pragmatic, backward-compatible evolution of existing cancer registry datasets could improve the quality, interoperability, and long-term usability of molecular real-world data. Outlining how structured, machine-readable variant documentation might fit within the current oBDS framework identifies a pathway that could enhance traceability, reduce ambiguity, and support secondary use in registries, quality assurance, and research.

Importantly, alignment with international standards, including HGVS-based nomenclature and explicit transcript and reference genome information, could support cross-border interoperability. In a European context, such data could support collaborative research infrastructures, distributed analytics, and emerging health data spaces, provided governance, interoperability, and technical implementation are addressed.

Looking ahead, a coordinated and standards-based evolution of the oncological core dataset is needed. Treating the oBDS as a living dataset, capable of controlled adaptation in response to scientific and technological advances, offers a pragmatic way to balance innovation, governance, interoperability, and data minimization. Achieving this will require sustained collaboration among cancer registries, pathology and laboratory medicine, clinical stakeholders, software providers, data protection experts, and regulatory institutions.

## Declaration of AI and AI-Assisted technologies in the writing process

During the preparation of this work, the authors used ChatGPT and Claude to improve readability and language. After using this tool/service, the authors reviewed and edited the content as needed and take full responsibility for the content of the publication.
